# The association between diabetes and abdominal aortic aneurysms in men: results of two Danish screening studies, a systematic review, and a meta-analysis of population-based screening studies

**DOI:** 10.1186/s12872-023-03160-8

**Published:** 2023-03-16

**Authors:** Katrine Lawaetz Larsen, Egle Kavaliunaite, Lars Melholt Rasmussen, Jesper Hallas, Axel Diederichsen, Flemming Hald Steffensen, Martin Busk, Lars Frost, Grazina Urbonaviciene, Jess Lambrechtsen, Kenneth Egstrup, Jes Sanddal Lindholt

**Affiliations:** 1 Department of Cardiac, Thoracic and Vascular Surgery, Odense University Hospital, J.B. Winsløws Vej 4, 5000 Odense C, Denmark; 2 The Danish Diabetes Academy, Odense University Hospital, Kløvervænget 6, 5000 Odense C, Denmark; 3 Department of Clinical Biochemistry and Pharmacology, Odense University Hospital, J.B. Winsløws Vej 4, 5000 Odense C, Denmark; 4 Clinical Pharmacology and Pharmacy, University of Southern Denmark, J.B. Winsløws Vej 19, 5000 Odense C, Denmark; 5 Department of Cardiology, Odense University Hospital, J.B. Winsløws Vej 4, 5000 Odense C, Denmark; 6 Department of Cardiology, Lillebaelt Hospital, Beriderbakken 4, 7100 Vejle, Denmark; 7 Department of Cardiology, Diagnostic Centre, Regional Hospital Silkeborg, Falkevej 1A, 8600 Silkeborg, Denmark; 8 Department of Cardiology, Odense University Hospital Svendborg, Baagøes Àlle 15, 5700 Svendborg, Denmark

**Keywords:** Ultrasound, Computerised tomography (CT), Meta-analysis, Abdominal aortic aneurysm, Screening

## Abstract

**Background:**

A paradoxical protective effect of diabetes on the development and progression of abdominal aortic aneurysms (AAA) has been known for years. This study aimed to investigate whether the protective role of diabetes on AAAs has evolved over the years.

**Methods:**

A cross-sectional study, a systematic review and meta-analysis. This study was based on two large, population-based, randomised screening trials of men aged 65–74; VIVA (2008–2011) and DANCAVAS (2014–2018), including measurement of the abdominal aorta by ultrasound or CT, respectively. Analyses were performed using multiple logistic regressions to estimate the odds ratios (ORs) for AAAs in men with diabetes compared to those not having diabetes. Moreover, a systematic review and meta-analysis of population-based screening studies of AAAs to visualise a potential change of the association between diabetes and AAAs. Studies reporting only on women or Asian populations were excluded.

**Results:**

In VIVA, the prevalence of AAA was 3.3%, crude OR for AAA in men with diabetes 1.04 (95% confidence interval, CI, 0.80-1.34), and adjusted OR 0.64 (CI 0.48-0.84). In DANCAVAS, the prevalence of AAA was 4.2%, crude OR 1.44 (CI 1.11-1.87), and adjusted OR 0.78 (CI 0.59-1.04). Twenty-three studies were identified for the meta-analysis (*N* = 224 766). The overall crude OR was 0.90 (CI 0.77-1.05) before 2000 and 1.16 (CI 1.03-1.30) after 1999. The overall adjusted OR was 0.63 (CI 0.59-0.69) before 2000 and 0.69 (CI 0.57-0.84) after 1999.

**Conclusion:**

Both the crude and adjusted OR showed a statistically non-significant trend towards an increased risk of AAA by the presence of diabetes. If this represents an actual trend, it could be due to a change in the diabetes population.

**Trial registration:**

DANCAVAS: Current Controlled Trials: ISRCTN12157806. VIVA: ClinicalTrials.gov NCT00662480.

**Supplementary Information:**

The online version contains supplementary material available at 10.1186/s12872-023-03160-8.

## Background

Traditionally, abdominal aortic aneurysms (AAAs) have been seen as a manifestation of atherosclerosis in the abdominal aorta, and the common risk factors for atherosclerosis, including diabetes, were assumed to apply [1, 2]. However, numerous studies have undermined the perception of AAAs as a manifestation of atherosclerosis and found that diabetes reduces the risk of AAA and its rate of progression [3].

The mechanism underlying the protective effect of diabetes has not been established. One study found an inverse association between fasting glucose and aortic diameter [4], while another did not find an association between fasting glucose and the presence of AAA [5].

We have previously shown that elevated glycated haemoglobin is associated with reduced AAA growth [6]. Studies found that antidiabetics in general and metformin are inversely associated with the presence and growth of AAAs [7–10]. On the other hand, we found no effect of long-term use of metformin regarding the risk of ruptured AAAs in a register-based study [11].

The definition of diabetes, the limits for fasting blood sugar, and the diagnostic tests have changed over the years [12]. Similarly, there is a change in the antidiabetic treatment [12, 13] and the prevention and treatment of other comorbidities, such as atherosclerosis [13]. Thus, the population with diabetes has changed over time, and the prevalence of type 2 diabetes has increased [13–15].

As mentioned, research has shown that elevated blood sugar could be an essential driver behind impaired growth and development of AAAs [4, 6]. Doctors are more aware of the consequences of poorly regulated diabetes, and glycated haemoglobin, reflecting average blood sugar over three months, might be lower on average now than 10–20 years ago [13]. People with diabetes benefit from this focus on reducing blood sugar, but it may influence the protective effect of diabetes on the development and progression of AAAs.

The present study aimed to investigate whether the protective role of diabetes on AAAs is consistent over time. Our objectives were to compare the prevalence of diabetes in men with and without AAAs in two large, Danish, population-based, randomised screening trials. Furthermore, to conduct a meta-analysis of screening studies measuring the abdominal aorta to compare odds ratios (ORs) for the association between AAA and diabetes. Thus, if aneurysmal growth is impaired by higher levels of glycated haemoglobin [6], we might see an effect on aneurysmal growth if people with diabetes have lower level of glycated haemoglobin now than 10–20 years ago [13]. The hypothesis was to demonstrate a shift from an inverse association between diabetes and AAAs to no association—or even an increased risk of AAA by the presence of diabetes.

## Methods

This cross-sectional study is based on two population-based, randomised, clinically controlled screening trials of men aged 65–74 years in the Central Denmark Region and the Region of Southern Denmark: VIVA [16, 17] and DANCAVAS [18, 19], respectively. Also, a meta-analysis of AAA screening studies preferably in men > 60 years.

### Data source

The Danish national healthcare system is tax-supported and provides the entire Danish population (5.8 million in 2019) with free, unrestricted access to public health services and partial reimbursement for most prescribed drugs. All Danish residents are assigned a unique 10-digit civil registration number at birth or immigration. Using the civil registration system, we randomly selected the participants in our screening trials based on sex, age, and the municipality of residence. The trial participation was free of charge.

#### The VIVA trial

The Viborg Vascular (VIVA) Screening Trial is a population-based, randomised, clinically controlled screening trial. Some 50 170 Danish men aged 65–74 years living in the Central Denmark Region were enrolled from 2008 to 2011 [16]. Briefly; study participants were randomly assigned 1:1 to triple screening or no systematic screening. Lifestyle parameters, medical history, and use of medication were self-reported. The triple screening included evaluation of AAA, hypertension, and peripheral arterial disease (PAD) with specially trained nurses performing ultrasound scans of the infrarenal abdominal aorta measuring the maximal systolic inner-to-inner anterior–posterior diameter. A maximal infrarenal aortic diameter ≥ 30 mm was defined as AAA. Dilatations ≥ 50 mm were referred for computed tomography (CT) followed by a consultation with a vascular surgeon if the aorta was ≥ 55 mm.

#### The DANCAVAS trial

The Danish Cardiovascular Screening Trial (DANCAVAS) is a population-based, randomised, clinically controlled screening trial with enrolment from 2014 to 2018 in the Region of Southern Denmark [18]. A total of 47 322 Danish men aged 65–74 years were randomised 1:2 to a seven-faceted cardiovascular screening or no systematic screening. The screening included a low-dose non-contrast CT to detect aortic/iliac aneurysms and coronary artery calcification. Furthermore, an evaluation of hypertension, PAD, and telemetric assessment of the heart rhythm. Lifestyle parameters, medical history, and use of medication were self-reported. The maximal outer-to-outer anterior–posterior diameter of the infrarenal aorta was measured, and aorta ≥ 30 mm was defined AAA. Dilatations ≥ 55 were referred for contrast-enhanced CT, followed by a consultation with a vascular surgeon.

### Covariates

Based on the questionnaires, we used self-reported diabetes or the use of antidiabetics in our analyses. Oral antidiabetics were not divided into subgroups. Body mass index (BMI) was classified as follows: < 18.5, underweight; 18.5–24.9, normal; 25–29.9, overweight; and ≥ 30, obese. In our analyses, we used measurements of the anterior–posterior diameter of the aorta.

### Inclusion

We defined our study population based on the source population. For both VIVA and DANCAVAS, the inclusion criterion was a measurement of the abdominal aorta.

### Analyses

We used baseline data of the VIVA and DANCAVAS populations to investigate whether the presence of AAA was associated with diabetes. Both simple and multiple logistic regression estimated the ORs for AAA and diabetes for both populations.

Group comparisons were made using either parametric or non-parametric tests. *P* < 0.05 was considered significant. Crude and adjusted ORs were calculated using simple and multiple logistic regression. The 95% confidence intervals (CIs) are given with the corresponding OR.

### Potential confounders

Questionnaires provided information on the participants' medical histories, smoking status, and current medications. In both populations, current medications and comorbidities were self-reported. PAD was defined as an ankle-brachial index < 0.9 or > 1.4 at the screening. In VIVA, we extracted previous acute myocardial infarction (AMI) and chronic obstructive pulmonary disease from the National Patient Register using the civil registration number. Medications were grouped according to the Anatomical Therapeutic Chemical (ATC) classification system developed by the World Health Organization [20]. Medications included oral blood-glucose-lowering medications (A10B), insulin (A10A), statins (C10AA), angiotensin-converting enzyme inhibitors (C09AA, C09BA, C09BB, C02EA, and C02LM), angiotensin II receptor antagonists (C09C), beta-blockers (C07), calcium antagonists (C08), low dose acetylsalicylic acid (B01AC06), non-steroidal anti-inflammatory drugs (M01AB), oral corticosteroids (H02), and inhaled corticosteroids (R03BA). All medications were categorised in groups, as mentioned above, and not specified by subgroup. Antidiabetic therapy was categorised in oral antidiabetics or insulins.

Potential confounders were selected based on an automated empirical procedure. We selected a wide range of variables and calculated the OR for having AAA and diabetes with or without the potential confounder included. If the OR for diabetes changed > 5% in either direction by adding the potential confounder, we added the variable in our multiple analysis. Thus, the following factors that empirically behaved as confounders were included in our model: a) age, b) smoking status, c) grouped BMI, d) presence of PAD, e) previous AMI, f) self-reported hypertension, g) use of statins, h) acetylsalicylic acid, i) beta-blockers, and j) angiotensin-converting enzyme inhibitors grouped with angiotensin II receptor antagonists.

### Meta-analysis

#### Literature extraction

Literature searches were performed in Embase, Medline, and Cochrane databases. KL conducted the search, and the last search was performed on November 16, 2018. The search terms were *abdominal aortic aneurysm* and *screening*, using both Medical Subject Headings and free text searches (see the supplementary material for the search strategy). These citations were searched manually to identify studies comprising abdominal aortic measurements with the prevalence of diabetes. In addition, the bibliographies from the citations as well as reviews were searched to identify additional studies.

#### Eligibility

We limited the articles to those written in English and included studies with original data regarding AAA screening and the presence of diabetes. Studies solely examining women were excluded, as the prevalence of AAA is different in men and women [2, 21]. Furthermore, studies with only Asian populations were excluded because the prevalence of both diabetes and AAA is reversed. The prevalence of AAA in Asian populations is up to tenfold lower [22], and the prevalence of diabetes is up to fourfold higher than Caucasians [23]. We excluded studies based on a select group of participants (e.g., a specific diagnosis) or a referral for outpatient evaluation (e.g., carotid or cardiac) concomitant with screening for AAAs. Furthermore, studies with self-referred participants or people participating due to some select membership and studies with information about AAAs based on hospital or discharge records were excluded. Thus, we only included population-based screenings to minimise the risk of bias. The outcome of interest was the OR with corresponding CI for having AAA and diabetes.

The eligibility assessment was performed independently by two authors, KL and EK, and a consensus was resolved. Data extraction was performed by KL and verified by EK. Extracted data included the year of study sampling, country, inclusion and exclusion criteria, number of participants, ethnicity, sex, age, the prevalence of AAA and diabetes, and the OR (crude and adjusted) for having AAA and diabetes with the corresponding CI. If possible, in studies with both men and women, we extracted data only for men. KL contacted eight authors of original studies for missing information; one responded but provided no additional information. A review protocol has not been published. PRISMA guidelines were used for the preparation and reporting of the meta-analysis [24].

#### Analyses

ORs with CIs from the individual studies were combined visually in a Forest plot sorted by data sampling time. If the year of inclusion was not given, we used the year of publication (*n* = 4). If an OR was not available, we calculated crude ORs based on the prevalence of diabetes in people with and without an AAA (*n* = 18). We performed a subgroup analysis by stratifying before and after the millennium. The measure of consistency, I [2], was reported.

All analyses were performed using Stata® Release 15.1 (StataCorp, College Station, TX, USA).

## Results

### Association between AAA and diabetes, VIVA

The VIVA population comprised 18 697 men with a median age of 69 years (Table 1). The participation rate was 74.7% [17]. The median aorta was 17.8 mm for men with diabetes and 18.3 mm for men without diabetes (*p* < 0.001). The prevalence of AAA was 3.3%.

**Table 1:** Characteristics of the VIVA and DANCAVAS population

**Characteristics**	*VIVA* + *DM*	*VIVA – DM*	*DANCAVAS* + *DM*	*DANCAVAS–DM*
**Men**	(*n* = 2027)	(*n* = 16 632)	(*n* = 1245)	(*n* = 9223)
**Age**	69 (67–71)	69 (67–71)	69 (67–71)	69 (67–71)
**Family history of AAA**	64 (3.2%)	538 (3.2%)	56 (4.5%)	432 (4.7%)
**BMI**				
< 25	365 (18.0%)	5709 (34.3%)	142 (11.4%)	2332 (25.3%)
≥ 25—< 30	935 (46.1%)	8159 (49.1%)	505 (40.6%)	4658 (50.5%)
≥ 30	700 (34.5%)	2535 (15.2%)	596 (47.9%)	2222 (24.1%)
**Smoking**				
Current	378 (18.6%)	3550 (21.3%)	197 (15.8%)	1385 (15.0%)
Former	1141 (56.3%)	8109 (48.8%)	718 (57.7%)	4804 (52.1%)
Never	505 (24.9%)	4963 (29.8%)	328 (26.3%)	3002 (32.5%)
**Abdominal aortic measurement**	17 (16–19)	18 (16–20)	19 (17–21)	19 (18–21)
**AAA**	69 (3.4%)	546 (3.3%)	71 (5.7%)	372 (4.0%)
**Self-reported comorbidity**				
Diabetes mellitus	2027 (100%)	-	1245 (100%)	-
Previous AMI	82 (4.0%)	411 (2.5%)	145 (11.6%)	548 (5.9%)
Hypertension	1424 (70.3%)	6529 (39.3%)	950 (76.3%)	3837 (41.6%)
COPD	70 (3.5%)	394 (2.4%)	104 (8.4%)	605 (6.6%)
PAD^a^	479 (23.6%)	2281 (13.7%)	278 (22.3%)	881 (9.6%)
**Drugs**				
Antidiabetics, oral	953 (47.0%)	-	895 (71.9%)	-
Insulin	452 (22.3%)	-	306 (24.6%)	-
Statins	1507 (74.3%)	5540 (33.3%)	949 (76.2%)	2787 (30.2%)
ACE inhibitors and ATII antagonists	1326 (65.4%)	4780 (28.7%)	852 (68.4%)	2827 (30.7%)
Beta-blockers	710 (35.0%)	3354 (20.2%)	393 (31.6%)	1343 (14.6%)
Acetylsalicylic acid	1259 (62.1%)	5192 (31.2%)	425 (34.1%)	1311 (14.2%)

Data are given as n (%) or median (interquartile range) unless otherwise noted

*DM* Diabetes mellitus, *AAA* Abdominal aortic aneurysm, *BMI* Body mass index, *AMI* Acute myocardial infarction, *COPD* Chronic obstructive pulmonary disease, *PAD* Peripheral arterial disease, *ACE* Angiotensin-converting enzyme, *ATII* Angiotensin II receptor

^a^Diagnosed by ankle-brachial index at the screening

Using logistic regression, we found a crude OR of 1.04 (95% CI 0.80–1.34) for having an AAA and diabetes compared to no diabetes. When adjusting for confounders, the OR reduced to 0.64 (CI 0.48–0.84). Age, BMI, smoking (both former and present), presence of hypertension and PAD, and the use of statins and acetylsalicylic acid were significant factors (Supplementary Table S1). Adjusting for the potential variables one at a time, we found that the use of statins shifted the OR towards a positive association and away from the crude OR more than the other potential confounders (Supplementary Table S2).

### Association between AAA and diabetes, DANCAVAS

The DANCAVAS population comprised 10 468 men with a median age of 69 years (Table 1). The participation rate was 62.4% [19]. The median aorta was 19.6 mm for men with diabetes and 19.8 mm for men without diabetes (*p* = 0.034). The prevalence of AAA was 4.2%.

Using logistic regression, we found a positive association between having AAA and diabetes compared to no diabetes (crude OR 1.44, CI 1.11–1.87). However, when adjusting for confounders, the OR reduced to 0.78 (CI 0.59–1.04). Age, BMI, smoking (both former and present), presence of hypertension and PAD, previous AMI, and the use of statins and acetylsalicylic acid were significant factors (Supplementary Table S1). Again, we found that adjusting for the use of statins distinctly shifted the OR compared to other potential confounders (Supplementary Table S2).

### Meta-analysis

**Fig. 1:**
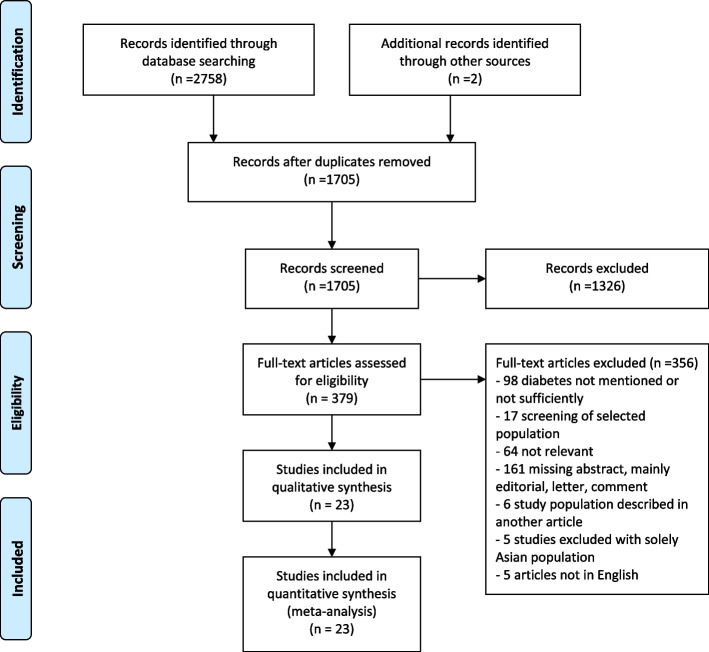
Prisma flow chart of studies in the meta-analysis

In Embase, Medline, and Cochrane databases, we identified 2758 articles and abstracts, 21 of which were relevant and assessed in detail [2, 4, 21, 25–42]. Moreover, we found two eligible studies reporting unique data by manual search [1, 43] (Fig. 1). Thus, 23 population-based studies were included in the review (Table 2 and Supplementary Table S3).

**Table 2:** Studies included in the meta-analysis; 23 studies and the results from our Danish studies

Study	Age	Men %	AAA/no AAA	AAA + DM/ AAA-DM	Crude OR (CI)	Adjusted OR (CI)	Ref
Brazil^a^ 1987–93	≥ 55	100^ h^	17/995	1/16	0.79 (0.10–6.04)	-	[27]
Italy 1991–94	65–75	46.3	70/1531	9/56	0.98 (0.48–2.02)	-	[37]
USA 1992–93	≥ 65	41.3^ h^	252/1701	40/212	0.84 (0.59–1.20)	-	[30]
USA^b^ 1992–95	50–79	97.2	2335/70085	NA	-	0.68 (0.60–0.77)	[33]
USA^c^ 1992–95	50–79	97.2	1031/70085	NA	-	0.54 (0.44–0.65)	[33]
England 1993	65–75	100^ h^	219/2378	11/208	0.83 (0.44–1.55)	-	[39]
Norway^d^ 1994–95	55–74	48.0^ h^	251/2335	7/244	0.69 (0.32–1.51)	-	[43]
Belgium 1995–96	65&75	100^ h^	33/694	7/26	2.13 (0.89–5.06)	-	[42]
USA^b^ 1995–97	50–79	97.4	1304/50828	NA	-	0.60 (0.50–0.71)	[21]
USA^c^ 1995–97	50–79	97.4	613/50828	NA	-	0.50 (0.39–0.65)	[21]
Netherlands^d^ 1995	≥ 55	42.0^ h^	91/2126	7/84	0.72 (0.33–1.57)	-	[1]
Australia 1996–99	65–79	100^ h^	933/11270	103/830	0.89 (0.72–1.11)	0.79 (0.63–0.98)	[4]
England 1996	65–80	43.4^ h^	178/2163	10/168	1.08 (0.56–2.10)	0.80 (0.41–1.58)	[2]
Scotland 2001–04	65–74	100^ h^	414/7732	43/371	0.93 (0.68–1.29)	-	[29]
Brazil^e^ 2002–03	≥ 60	34.3	21/806	5/16	1.63 (0.59–4.51)	-	[26]
Sweden^f^ 2006–10	65	100^ h^	233/14378	24/209	0.83 (0.54–1.26)	-	[41]
Sweden 2007–07	65–75	100^ h^	168/14081	10/158	1.31 (0.69–2.50)	-	[40]
Italy 2007–09	≥ 65	52.6	512/7722	66/401	1.41 (1.07–1.84)	-	[35]
Spain 2007–10	65–74	100^ h^	15/636	3/12	0.77 (0.21–2.76)	-	[36]
Spain^g^ 2008–09	65	100^ h^	37/739	NA	0.28 (0.08–0.90)	0.38 (0.11–1.06)	[25]
Sweden 2008–10	70	100^ h^	107/4608	19/88	1.19 (0.72–1.96)	-	[32]
Denmark 2008–11	64–75	100^ h^	617/18080	69/546	1.04 (0.80–1.34)	0.64 (0.48–0.84)	
Italy 2010–13	60–85	48.6^ h^	19/735	1/18	0.35 (0.05–2.64)	-	[28]
Spain 2013–14	≥ 60	100^ h^	11/998	3/8	1.00 (0.26–3.80)	-	[38]
Italy 2013–16	50–75	63.7^ h^	56/2335	11/45	1.51 (0.77–2.95)	-	[31]
Belgium 2014–14	65–85	65.6^ h^	35/687	7/28	1.06 (0.45–2.48)	-	[34]
Denmark 2014–18	65–74	100^ h^	443/10025	71/372	1.44 (1.11–1.87)	0.78 (0.59–1.04)	

*AAA* Abdominal aortic aneurysm, *DM* Diabetes, *OR* Odds ratio, *CI* Confidence interval, *Ref* Reference number

^a^A study of three groups, the reference group based on the general population is depicted

^b^Aorta 30–39 mm compared to < 30 mm

^c^Aorta ≥ 40 mm compared to < 30 mm

^d^Extra by manual search. Both studies defined AAA as aorta ≥ 35 mm

^e^Different number in their table (total diabetes) compared to the total number of 834

^f^Only data on 14,611 despite another number given

^g^Unable to measure aorta in 5

^h^Prevalence and OR in men only

The studies were conducted between 1987 and 2018. The majority of the studies did not report crude or adjusted ORs for AAA and diabetes, but we calculated crude ORs by extracting the prevalence. Moreover, 11 studies (47.8%) included only men, but crude ORs were calculated for men in 15 studies (65.2%) based on the reported numbers.

**Fig. 2:**
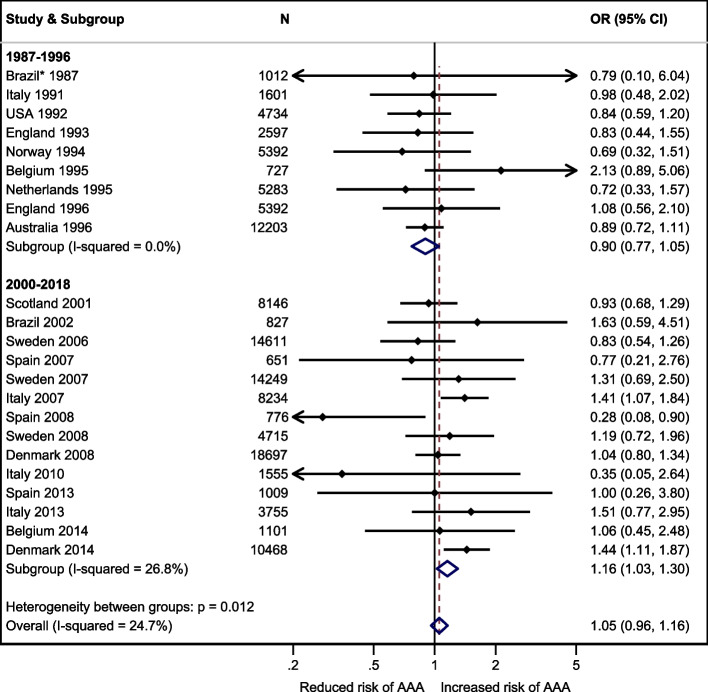
Forest plot of the crude OR of having an AAA and diabetes compared to no diabetes sorted by year of inclusion and a subgroup analysis before and after the millennium. *A study of three groups, the reference group based on the general population is depicted

**Fig. 3:**
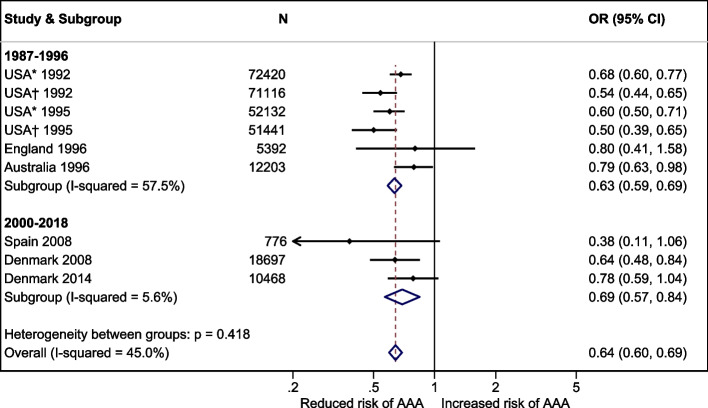
Forest plot of the adjusted OR of having an AAA and diabetes compared to no diabetes sorted by year of inclusion and a subgroup analysis before and after the millennium. *The authors compared aorta 30–39 mm with no aneurysm; †The authors compared AAA ≥ 40 mm with no aneurysm

In the meta-analysis, we found a change in the crude OR over time, as visualised in Fig. 2. However, substantial confounding was corrected, and the adjusted OR remained almost unchanged over time (Fig. 3).

## Discussion

In this observational study of 29 165 men aged 65–74 years in whom the association between AAA and diabetes was estimated, we found a significant inverse association in the VIVA population (2008–2011), but not in the later DANCAVAS population (2014–2018).

In VIVA, after adjusting for potential confounders, we found that the presence of diabetes significantly reduced the OR for AAA by 38%. In DANCAVAS, in the crude analysis, men with diabetes had almost 50% higher odds of having an AAA compared to no diabetes. When adjusting for potential confounders, the inverse association was not significant. Overall, the use of statins affected the OR towards a positive association, which could be due to the use of statins per se or an indirect adjustment for high cardiovascular risk. Previous studies have found conflicting results regarding the use of statins, finding either no effect on growth rate [7, 44] or a reduced growth rate of AAA [45]. Furthermore, studies have observed an increased risk of AAA with the presence of atherosclerosis, including claudication [1] and coronary artery disease [41].

The results of our two Danish screening trials could indicate a shift in the positive association between diabetes and AAA. Over time, there has been a change in the prevalence of AAA-associated risk factors (Table 1). An independent positive association seems to persist after adjusting for these dispositions in VIVA, but not DANCAVAS. The meta-analysis found a similar shift over time towards a change in the risk of having an AAA and diabetes. However, the independent protective effect of diabetes on AAAs remained unchanged. When we compared the adjusted analyses of the studies included in the meta-analysis, we found that none was adjusted for statins. However, they were adjusted to some form of atherosclerosis (Table S3). It is important to emphasise that the definition of diabetes, diagnostic tests, and antidiabetic therapy have improved over the years [12–15]. The prevalence of people with diabetes has increased [14, 46], and the management, resulting in an increased prevalence of people receiving antidiabetic therapy such as metformin [13, 15]. Consequently, there could be an earlier diagnosis and improved glycaemic and risk profiles resulting in less severe and better-managed diabetes in the later studies than in the earlier studies.

Studies have found a decreased prevalence of AAAs [47] with the use of metformin and a reduced growth rate [8–10]. It is impossible to deduce anything regarding metformin from this study since we do not have information about the subgroups of oral antidiabetic therapy.

### Strengths and limitations

Our study has some strengths. First, the data are based on two large, population-based, randomised, clinically controlled screening trials with 29 165 male participants. One trial comprises ultrasound-verified AAAs and the other non-contrast CT-verified AAAs. As the participants were randomly selected according to their unique civil registration number and participation rates were high, the risk of selection bias was minimal. On the other hand, we cannot reject selection bias, as people with severe diabetes, or any severe disease in general, may be unwilling to attend voluntary screenings [48, 49]. In our study, the prevalence of diabetes was 11.0% for VIVA (launched in 2008). In a study based on the Danish National Diabetes Register, the prevalence of diabetes was approximately 15% for men aged 70 years [50]. Thus, we have an underrepresentation of men with diabetes, which could shift the OR towards or above 1. However, suppose we have an underrepresentation of men with severe diabetes. In that case, other population-based studies may as well, and, therefore, our results are comparable when we look at the shift over time. Our meta-analysis was comprehensive with only two search terms, *abdominal aortic aneurysm* and *screening*. We found several studies not mentioning diabetes at all except in the methods and a table. We assumed that the risk of measurement error across studies was minor, as screening for AAAs with ultrasound is reasonably reproducible in skilled hands. We took precautions regarding the meta-analysis and applied several exclusion criteria to minimise the risk of bias across studies. We excluded numerous studies based on a selected group of participants because we wanted a representative cross-section of the general population. There are several AAA screening studies in patient populations undergoing, for example, coronary artery bypass grafting. We excluded studies of Asian populations because Asian and Caucasian populations are not comparable in both AAA and diabetes [22, 23]. Lastly, we excluded studies with self-referred people because this may increase the risk of bias, as, for example, self-referred people are likely better educated or wealthier, and people with relatives with an AAA may be more prone to attend screenings for AAA. First degree relatives are well-known as having a higher risk of AAA [4, 21].

Our study has several potential limitations. Our data are based on self-reported information regarding lifestyle, medication, and previous illnesses and could, therefore, contain inaccuracies and recall bias, leading to misclassifications. We did not combine the two datasets in our analyses. The studies used different types of imaging for diagnosing AAA, and the prevalence of AAAs may differ between VIVA and DANCAVAS due to the nature of the screenings. The DANCAVAS screening was more comprehensive than the VIVA screening, and people may have attended despite a known diagnosis of AAA. Regarding generalizability, we only included men in our Danish screening trials, and the interpretation must be restricted to men. Furthermore, although the data originated from a clinical trial, the data should be regarded as observational, entailing a risk of confounding as addressed in this discussion. We tried to eliminate bias by doing the systematic review with broad search terms and not including "diabetes". Studies with positive findings of diabetes are reported in the abstract, but the broad search made it possible to include studies that focused elsewhere. Given that most of the studies included in our meta-analysis investigated the prevalence of AAAs, we assume the risk of publication bias or selective reporting is minimal. However, some screening studies do not report the prevalence of diabetes in detail. Another potential limitation is the definition of AAA. Most studies defined AAA as aorta ≥ 30 mm, some with a ratio ≥ 1.5 (infrarenal/suprarenal), whereas some defined it as aorta ≥ 35–40 mm. There is a risk of information bias since we sorted the studies by publication if the year of inclusion was missing. Our meta-analysis comprised population-based screening studies, which have the advantage of low expenses and are often faster to complete. However, they cannot exclude potential confounding factors, and we cannot conclude anything about causality. On the other hand, we focused solely on population-based studies based on the general population, i.e., every citizen could be included if he or she were the right age at the given time. Therefore, our results are based on a representative cross-section of the population, both the meta-analysis and the screening trials. We found some heterogeneity in our meta-analysis. However, we have included several studies in the analysis, and we mostly have overlap in the CI’s and with some CI’s rather wide. By including adjusted estimates in our model, we tried to eliminate the effect of potential confounders. However, there is a risk of residual confounding. The comparison of adjusted ORs in our meta-analysis carries some uncertainty. The studies do not adjust for precisely the same potential confounders, and data are based on a small number of studies. However, we added the potential confounders included in the studies in Table S1. Lastly, we estimated the association between AAA and diabetes, but in this study, it is impossible to know which appeared first; the AAA or diabetes.

## Conclusion

Both the crude and adjusted OR showed a statistically non-significant trend towards an increased risk of AAA by the presence of diabetes. If this represents an actual trend, it could be due to a change in the diabetes population.

## Supplementary Information

**Additional file 1: Table S1.** Adjusted odds ratio of the association between diabetes and abdominal aortic aneurysms with 95% confidence intervals and the potential confounders.**Table S2.** Crude and adjusted odds ratios (ORs) of the association between diabetes and abdominal aortic aneurysms with 95% confidence intervals. **Table S3.**Studies included in the meta-analysis; 23 studies identified in databases and the results from our Danish studies.

## Data Availability

The data that support the findings of this study are available from the corresponding author upon reasonable request. We did not preregister this study in an independent, institutional register. All authors had complete access to the study data supporting this publication.

## Abbreviations

AAA: Abdominal aortic aneurysm
ABI: Ankle-brachial index
AMI: Acute myocardial infarction
ATC: Anatomical Therapeutic Chemical classification system
BMI: Body mass index
CI: Confidence interval
COPD: Chronic obstructive pulmonary disease
CT: Computed tomography
DANCAVAS: The Danish Cardiovascular Screening Trial
OR: Odds ratio
PAD: Peripheral arterial disease
VIVA: Viborg Vascular Screening Trial
