# Supplementary material for: The association between diabetes and abdominal aortic aneurysms in men: results of two Danish screening studies, a systematic review, and a meta-analysis of population-based screening studies
Source: BMC Cardiovasc Disord. 2023 Mar 16;23:139. doi: 10.1186/s12872-023-03160-8 (PMC10022183; doi:10.1186/s12872-023-03160-8)
Supplement: Supplementary file 1 — Additional file 1: Table S1. Adjusted odds ratio of the association between diabetes and abdominal aortic aneurysms with 95% confidence intervals and the potential confounders.Table S2. Crude and adjusted odds ratios (ORs) of the association between diabetes and abdominal aortic aneurysms with 95% confidence intervals. Table S3.Studies included in the meta-analysis; 23 studies identified in databases and the results from our Danish studies. [file 12872_2023_3160_MOESM1_ESM.docx]

**Supplementary material**

**Search strategy**

**Databases:** Medline, Embase, and Cochrane. **Date of search:** November 16, 2018.

**Search string for Medline**

1. exp Aortic Aneurysm, Abdominal/

2. limit 1 to English language

3. abdominal aorta aneurysm*.mp. [mp=title, abstract, original title, name of substance word, subject heading word, floating sub-heading word, keyword heading word, protocol supplementary concept word, rare disease supplementary concept word, unique identifier, synonyms]

4. limit 3 to English language

5. abdominal aortic aneurysm*.mp. [mp=title, abstract, original title, name of substance word, subject heading word, floating sub-heading word, keyword heading word, protocol supplementary concept word, rare disease supplementary concept word, unique identifier, synonyms]

6. limit 5 to English language

7. abdominal aorta.mp. [mp=title, abstract, original title, name of substance word, subject heading word, floating sub-heading word, keyword heading word, protocol supplementary concept word, rare disease supplementary concept word, unique identifier, synonyms]

8. limit 7 to English language

9. 1 and 2

10. 3 and 4

11. 5 and 6

12. 7 and 8

13. 9 or 10 or 11 or 12

14. mass screening/ or multiphasic screening/

15. limit 14 to English language

16. screen*.mp. [mp=title, abstract, original title, name of substance word, subject heading word, floating sub-heading word, keyword heading word, protocol supplementary concept word, rare disease supplementary concept word, unique identifier, synonyms]

17. limit 16 to English language

18. 14 and 15

19. 16 and 17

20. 18 or 19

21. 13 and 20

**Table S1.** Adjusted odds ratio of the association between diabetes and abdominal aortic aneurysms with 95% confidence intervals and the potential confounders.

|  | VIVA | DANCAVAS |
| --- | --- | --- |
| Diabetes | 0.64 (0.48-0.84) | 0.78 (0.59-1.04) |
| Age | 1.06 (1.03-1.09) | 1.11 (1.07-1.15) |
| Former smoker | 3.23 (2.36-4.41) | 3.14 (2.23-4.42) |
| Current smoker | 6.89 (5.0-9.51) | 8.43 (5.88-12.09) |
| Overweight | 1.39 (1.13-1.70) | 1.13 (0.86-1.48) |
| Obese | 1.56 (1.21-2.01) | 1.59 (1.19-2.13) |
| Previous AMI | 1.43-0.97-2.11) | 2.07 (1.52-2.83) |
| Hypertension | 1.27 (1.03-1.57) | 1.39 (1.08-1.79) |
| PAD | 1.68 (1.38-2.05) | 1.70 (1.33-2.17) |
| Statins | 1.56 (1.27-1.93) | 1.85 (1.46-2.35) |
| ACE & ATII | 0.99 (0.80-1.22) | 0.97 (0.76-1.25) |
| Beta-blocker | 1.16 (0.94-1.43) | 0.88 (0.68-1.14) |
| Acetylsalicylic acid | 1.29 (1.04-1.60) | 1.37 (1.07-1.74) |

AAA, abdominal aortic aneurysm; AMI, acute myocardial infarction; PAD, peripheral arterial disease; ACE, angiotensin-converting enzyme inhibitor; ATII, angiotensin II receptor antagonists.

**Table S2.** Crude and adjusted odds ratios (ORs) of the association between diabetes and abdominal aortic aneurysms with 95% confidence intervals

|  | VIVA | DANCAVAS |
| --- | --- | --- |
| Crude OR | 1.04 (0.80-1.34) | 1.44 (1.11-1.87) |
| Adjusted OR age | 1.03 (0.80-1.33) | 1.42 (1.09-1.84) |
| Adjusted OR smoking | 1.03 (0.80-1.33) | 1.37 (1.05-1.78) |
| Adjusted OR BMI | 0.95 (0.73-1.24) | 1.29 (0.99-1.69) |
| Adjusted OR AMI | 1.02 (0.79-1.31) | 1.29 (0.99-1.68) |
| Adjusted OR hypertension | 0.88 (0.68-1.14) | 1.19 (0.91-1.55) |
| Adjusted OR PAD | 0.92 (0.71-1.19) | 1.25 (0.96-1.64) |
| Adjusted OR statin | 0.73 (0.56-0.95) | 0.91 (0.70-1.20) |
| Adjusted OR ACE & ATII | 0.89 (0.69-1.16) | 1.19 (0.90-1.55) |
| Adjusted OR beta-blocker | 0.93 (0.72-1.21) | 1.28 (0.98-1.66) |
| Adjusted OR acetylsalicylic acid | 0.80 (0.61-1.04) | 1.15 (0.88-1.50) |
| Adjusted OR all | 0.64 (0.48-0.84) | 0.78 (0.59-1.04) |

The ORs were adjusted by one variable at a time. BMI, body mass index; AMI, acute myocardial infarction; PAD, peripheral arterial disease; ACE, angiotensin-converting enzyme inhibitor; ATII, angiotensin II receptor antagonists.

**Table S3.** Studies included in the meta-analysis; 23 studies identified in databases and the results from our Danish studies.

| Study | Age | Men % | AAA/no AAA | AAA+DM/ AAA-DM | Crude OR | Adjusted OR | Ref |
| --- | --- | --- | --- | --- | --- | --- | --- |
| Brazil* 1987-93 | ≥55 | 100^##^ | 17/995 | 1/16 | 0.79 (0.10-6.04) | - | ^1^ |
| Italy 1991-94 | 65-75 | 46.3 | 70/1531 | 9/56 | 0.98 (0.48-2.02) | - | ^2^ |
| USA 1992-93 | ≥65 | 41.3^##^ | 252/1701 | 40/212 | 0.84 (0.59-1.20) | - | ^3^ |
| USA^†^ 1992-95 | 50-79 | 97.2 | 2335/70085 | NA | - | 0.68 (0.60-0.77) | ^4^ |
| USA^‡^ 1992-95 | 50-79 | 97.2 | 1031/70085 | NA | - | 0.54 (0.44-0.65) | ^4^ |
| England 1993 | 65-75 | 100^##^ | 219/2378 | 11/208 | 0.83 (0.44-1.55) | - | ^5^ |
| Norway^§^ 1994-95 | 55-74 | 48.0^##^ | 251/2335 | 7/244 | 0.69 (0.32-1.51) | - | ^6^ |
| Belgium 1995-96 | 65&75 | 100^##^ | 33/694 | 7/26 | 2.13 (0.89-5.06) | - | ^7^ |
| USA^†^ 1995-97 | 50-79 | 97.4 | 1304/50828 | NA | - | 0.60 (0.50-0.71) | ^8^ |
| USA^‡^ 1995-97 | 50-79 | 97.4 | 613/50828 | NA | - | 0.50 (0.39-0.65) | ^8^ |
| Netherlands^§^ 1995 | ≥55 | 42.0^##^ | 91/2126 | 7/84 | 0.72 (0.33-1.57) | - | ^9^ |
| Australia^¶^ 1996-99 | 65-79 | 100^##^ | 933/11270 | 103/830 | 0.89 (0.72-1.11) | 0.79 (0.63-0.98) | ^10^ |
| England^#^ 1996 | 65-80 | 43.4^##^ | 178/2163 | 10/168 | 1.08 (0.56-2.10) | 0.80 (0.41-1.58) | ^11^ |
| Scotland 2001-04 | 65-74 | 100^##^ | 414/7732 | 43/371 | 0.93 (0.68-1.29) | - | ^12^ |
| Brazil** 2002-03 | ≥60 | 34.3 | 21/806 | 5/16 | 1.63 (0.59-4.51) | - | ^13^ |
| Sweden^††^ 2006-10 | 65 | 100^##^ | 233/14378 | 24/209 | 0.83 (0.54-1.26) | - | ^14^ |
| Sweden 2007-07 | 65-75 | 100^##^ | 168/14081 | 10/158 | 1.31 (0.69-2.50) | - | ^15^ |
| Italy 2007-09 | ≥65 | 52.6 | 512/7722 | 66/401 | 1.41 (1.07-1.84) | - | ^16^ |
| Spain 2007-10 | 65-74 | 100^##^ | 15/636 | 3/12 | 0.77 (0.21-2.76) | - | ^17^ |
| Spain^‡‡^ 2008-09 | 65 | 100^##^ | 37/739 | NA | 0.28 (0.08-0.90) | 0.38 (0.11-1.06) | ^18^ |
| Sweden 2008-10 | 70 | 100^##^ | 107/4608 | 19/88 | 1.19 (0.72-1.96) | - | ^19^ |
| Denmark^§§^ 2008-11 | 64-75 | 100^##^ | 617/18080 | 69/546 | 1.04 (0.80-1.34) | 0.64 (0.48-0.84) |  |
| Italy 2010-13 | 60-85 | 48.6^##^ | 19/735 | 1/18 | 0.35 (0.05-2.64) | - | ^20^ |
| Spain 2013-14 | ≥60 | 100^##^ | 11/998 | 3/8 | 1.00 (0.26-3.80) | - | ^21^ |
| Italy 2013-16 | 50-75 | 63.7^##^ | 56/2335 | 11/45 | 1.51 (0.77-2.95) | - | ^22^ |
| Belgium 2014-14 | 65-85 | 65.6^##^ | 35/687 | 7/28 | 1.06 (0.45-2.48) | - | ^23^ |
| Denmark^§§^ 2014-18 | 65-74 | 100^##^ | 443/10025 | 71/372 | 1.44 (1.11-1.87) | 0.78 (0.59-1.04) |  |

AAA, abdominal aortic aneurysm; DM, diabetes; Ref, reference number.
*A study of three groups, the reference group based on the general population is depicted. ^†^Aorta 30-39 mm compared to <30 mm. Adjusted for age, sex, ethnicity, height, weight, waist circumference, family history of AAA, history of smoking, hypertension, high cholesterol, coronary artery disease (CAD), claudication, cerebral vascular disease (CVD), deep venous thrombosis (DVT), diabetes, chronic obstructive pulmonary disease (COPD), non-skin cancer, and abdominal imaging in past five years. ^‡^Aorta≥40 mm compared to <30 mm, adjusted for the same as mentioned before. ^§^Extra by manual search. Both studies defined AAA as aorta ≥35 mm. ^¶^Adjusted for age, body mass index (BMI), place of birth, smoking, diastolic and systolic blood pressure, hypertension, dyslipidaemia, diabetes, family history of AAA, vigorous exercise, and history of CAD, CVD, peripheral arterial disease (PAD) and mesenteric artery disease. ^#^Adjusted for age, sex, smoking, respiratory disease, acute myocardial infarction (AMI), angina, stroke, diabetes, and claudication. **Different number in their table (total diabetes) compared to the total number of 834. ^††^Only data on 14,611 despite another number given. ^‡‡^Unable to measure aorta in 5. Adjusted for family history of AAA, smoking, DM, hypertension, chronic renal failure, CVD, and PAD. ^§§^Adjusted for age, smoking status, grouped BMI, presence of PAD, previous AMI, hypertension, and use of statins, acetylsalicylic acid, beta-blockers, and angiotensin-converting enzyme inhibitors plus angiotensin II receptor antagonists. ^##^Prevalence and OR in men only.

**Prisma checklist**

| **Section/topic** | **#** | **Checklist item** | **Reported on page #** |
| --- | --- | --- | --- |
| **TITLE** | | | |
| Title | 1 | Identify the report as a systematic review, meta-analysis, or both. | 1 |
| **ABSTRACT** | | | |
| Structured summary | 2 | Provide a structured summary including, as applicable: background; objectives; data sources; study eligibility criteria, participants, and interventions; study appraisal and synthesis methods; results; limitations; conclusions and implications of key findings; systematic review registration number. | 4 |
| **INTRODUCTION** | | | |
| Rationale | 3 | Describe the rationale for the review in the context of what is already known. | 5-6 |
| Objectives | 4 | Provide an explicit statement of questions being addressed with reference to participants, interventions, comparisons, outcomes, and study design (PICOS). | 6 |
| **METHODS** | | | |
| Protocol and registration | 5 | Indicate if a review protocol exists, if and where it can be accessed (e.g., Web address), and, if available, provide registration information including registration number. | 10 |
| Eligibility criteria | 6 | Specify study characteristics (e.g., PICOS, length of follow-up) and report characteristics (e.g., years considered, language, publication status) used as criteria for eligibility, giving rationale. | 9-10 |
| Information sources | 7 | Describe all information sources (e.g., databases with dates of coverage, contact with study authors to identify additional studies) in the search and date last searched. | 9-10 |
| Search | 8 | Present full electronic search strategy for at least one database, including any limits used, such that it could be repeated. | Supplementary |
| Study selection | 9 | State the process for selecting studies (i.e., screening, eligibility, included in systematic review, and, if applicable, included in the meta-analysis). | 9-10 |
| Data collection process | 10 | Describe method of data extraction from reports (e.g., piloted forms, independently, in duplicate) and any processes for obtaining and confirming data from investigators. | 9-10 |
| Data items | 11 | List and define all variables for which data were sought (e.g., PICOS, funding sources) and any assumptions and simplifications made. | 9-10 |
| Risk of bias in individual studies | 12 | Describe methods used for assessing risk of bias of individual studies (including specification of whether this was done at the study or outcome level), and how this information is to be used in any data synthesis. | 9-10 |
| Summary measures | 13 | State the principal summary measures (e.g., risk ratio, difference in means). | 10 |
| Synthesis of results | 14 | Describe the methods of handling data and combining results of studies, if done, including measures of consistency (e.g., I^2^) for each meta-analysis. | 10 |
| Risk of bias across studies | 15 | Specify any assessment of risk of bias that may affect the cumulative evidence (e.g., publication bias, selective reporting within studies). | 10 + 13-15 |
| Additional analyses | 16 | Describe methods of additional analyses (e.g., sensitivity or subgroup analyses, meta-regression), if done, indicating which were pre-specified. | 10 |
| **RESULTS** | | | |
| Study selection | 17 | Give numbers of studies screened, assessed for eligibility, and included in the review, with reasons for exclusions at each stage, ideally with a flow diagram. | 12 + figure 1 |
| Study characteristics | 18 | For each study, present characteristics for which data were extracted (e.g., study size, PICOS, follow-up period) and provide the citations. | Table 2 + S2 |
| Risk of bias within studies | 19 | Present data on risk of bias of each study and, if available, any outcome level assessment (see item 12). | Table 2 + S2 |
| Results of individual studies | 20 | For all outcomes considered (benefits or harms), present, for each study: (a) simple summary data for each intervention group (b) effect estimates and confidence intervals, ideally with a forest plot. | Figure 2-3 |
| Synthesis of results | 21 | Present the main results of the review. If meta-analyses are done, include for each, confidence intervals and measures of consistency. | 12-13 + figure 2-3 |
| Risk of bias across studies | 22 | Present results of any assessment of risk of bias across studies (see Item 15). | 13-15 |
| Additional analysis | 23 | Give results of additional analyses, if done (e.g., sensitivity or subgroup analyses, meta-regression [see Item 16]). | 12 + figure 2-3 |
| **DISCUSSION** | | | |
| Summary of evidence | 24 | Summarize the main findings including the strength of evidence for each main outcome; consider their relevance to key groups (e.g., healthcare providers, users, and policy makers). | 13-15 |
| Limitations | 25 | Discuss limitations at study and outcome level (e.g., risk of bias), and at review-level (e.g., incomplete retrieval of identified research, reporting bias). | 13-15 |
| Conclusions | 26 | Provide a general interpretation of the results in the context of other evidence, and implications for future research. | 13-15 |
| **FUNDING** | | | |
| Funding | 27 | Describe sources of funding for the systematic review and other support (e.g., supply of data); role of funders for the systematic review. | 18 |

*From:*  Moher D, Liberati A, Tetzlaff J, Altman DG, The PRISMA Group (2009). Preferred Reporting Items for Systematic Reviews and Meta-Analyses: The PRISMA Statement. PLoS Med 6(7): e1000097. doi:10.1371/journal.pmed100

**References**

1. Bonamigo TP, Siqueira I. Screening for abdominal aortic aneurysms. Rev Hosp Clin Fac Med Sao Paulo. 2003;58(2):63-8.

2. Simoni G, Pastorino C, Perrone R, Ardia A, Gianrossi R, De Cian F, et al. Screening for abdominal aortic aneurysms and associated risk factors in a general population. Eur J Vasc Endovasc Surg. 1995;10(2):207-10.

3. Freiberg MS, Arnold AM, Newman AB, Edwards MS, Kraemer KL, Kuller LH. Abdominal aortic aneurysms, increasing infrarenal aortic diameter, and risk of total mortality and incident cardiovascular disease events: 10-year follow-up data from the Cardiovascular Health Study. Circulation. 2008;117(8):1010-7.

4. Lederle FA, Johnson GR, Wilson SE, Chute EP, Littooy FN, Bandyk D, et al. Prevalence and associations of abdominal aortic aneurysm detected through screening. Aneurysm Detection and Management (ADAM) Veterans Affairs Cooperative Study Group. Ann Intern Med. 1997;126(6):441-9.

5. Smith FC, Grimshaw GM, Paterson IS, Shearman CP, Hamer JD. Ultrasonographic screening for abdominal aortic aneurysm in an urban community. Br J Surg. 1993;80(11):1406-9.

6. Jorgensen L, Singh K, Berntsen GK, Jacobsen BK. A population-based study of the prevalence of abdominal aortic aneurysms in relation to bone mineral density: the Tromso study. Am J Epidemiol. 2004;159(10):945-9.

7. Vazquez C, Sakalihasan N, D'Harcour JB, Limet R. Routine ultrasound screening for abdominal aortic aneurysm among 65- and 75-year-old men in a city of 200,000 inhabitants. Ann Vasc Surg. 1998;12(6):544-9.

8. Lederle FA, Johnson GR, Wilson SE, Chute EP, Hye RJ, Makaroun MS, et al. The aneurysm detection and management study screening program: validation cohort and final results. Aneurysm Detection and Management Veterans Affairs Cooperative Study Investigators. Arch Intern Med. 2000;160(10):1425-30.

9. Pleumeekers HJ, Hoes AW, van der Does E, van Urk H, Hofman A, de Jong PT, et al. Aneurysms of the abdominal aorta in older adults. The Rotterdam Study. Am J Epidemiol. 1995;142(12):1291-9.

10. Le MT, Jamrozik K, Davis TM, Norman PE. Negative association between infra-renal aortic diameter and glycaemia: the Health in Men Study. Eur J Vasc Endovasc Surg. 2007;33(5):599-604.

11. Kanagasabay R, Gajraj H, Pointon L, Scott RA. Co-morbidity in patients with abdominal aortic aneurysm. J Med Screen. 1996;3(4):208-10.

12. Duncan JL, Harrild KA, Iversen L, Lee AJ, Godden DJ. Long term outcomes in men screened for abdominal aortic aneurysm: prospective cohort study. BMJ. 2012;344:e2958.

13. Barros F, Pontes S, Taylor M, Roelke L, Sandri J, De Melo Jacques C, et al. Screening for abdominal aortic aneurysm in the population of the city of Vitoria, ES, Brazil. J Vasc Bras. 2005;4(1):59-65.

14. Svensjo S, Bjorck M, Gurtelschmid M, Djavani Gidlund K, Hellberg A, Wanhainen A. Low prevalence of abdominal aortic aneurysm among 65-year-old Swedish men indicates a change in the epidemiology of the disease. Circulation. 2011;124(10):1118-23.

15. Stackelberg O, Wolk A, Eliasson K, Hellberg A, Bersztel A, Larsson SC, et al. Lifestyle and Risk of Screening-Detected Abdominal Aortic Aneurysm in Men. J Am Heart Assoc. 2017;6(5):e004725.

16. Palombo D, Lucertini G, Pane B, Mazzei R, Spinella G, Brasesco PC. District-based abdominal aortic aneurysm screening in population aged 65 years and older. The Journal of cardiovascular surgery. 2010;51(6):777-82.

17. Salvador-Gonzalez B, Martin-Baranera M, Borque-Ortega A, Saez-Saez RM, de Albert-Delas Vigo M, Carreno-Garcia E, et al. Prevalence of Abdominal Aortic Aneurysm in Men Aged 65-74 Years in a Metropolitan Area in North-East Spain. Eur J Vasc Endovasc Surg. 2016;52(1):75-81.

18. Barba A, Vega de Ceniga M, Estallo L, de la Fuente N, Viviens B, Izagirre M. Prevalence of abdominal aortic aneurysm is still high in certain areas of southern Europe. Ann Vasc Surg. 2013;27(8):1068-73.

19. Hager J, Lanne T, Carlsson P, Lundgren F. Lower prevalence than expected when screening 70-year-old men for abdominal aortic aneurysm. Eur J Vasc Endovasc Surg. 2013;46(4):453-9.

20. Corrado G, Durante A, Genchi V, Trabattoni L, Beretta S, Rovelli E, et al. Prevalence of previously undiagnosed abdominal aortic aneurysms in the area of Como: the ComoCuore "looking for AAA" ultrasonography screening. Int J Cardiovasc Imaging. 2016;32(8):1213-7.

21. Siso-Almirall A, Kostov B, Navarro Gonzalez M, Cararach Salami D, Perez Jimenez A, Gilabert Sole R, et al. Abdominal aortic aneurysm screening program using hand-held ultrasound in primary healthcare. PLoS ONE. 2017;12(4):e0176877.

22. Gianfagna F, Veronesi G, Tozzi M, Tarallo A, Borchini R, Ferrario MM, et al. Prevalence of Abdominal Aortic Aneurysms in the General Population and in Subgroups at High Cardiovascular Risk in Italy. Results of the RoCAV Population Based Study. Eur J Vasc Endovasc Surg. 2018;55(5):633-9.

23. Makrygiannis G, Labalue P, Erpicum M, Schlitz M, Seidel L, El Hachemi M, et al. Extending Abdominal Aortic Aneurysm Detection to Older Age Groups: Preliminary Results from the Liege Screening Programme. Ann Vasc Surg. 2016;36:55-63.
